# Prevalence of Anxiety and Depression and Their Association With Migraine Among PHC Center Visitors in Madina, Saudi Arabia

**DOI:** 10.7759/cureus.55404

**Published:** 2024-03-02

**Authors:** Mohammed Qarah, Noura Alshammari, Rafa Alsharif, Manal Albalawi, Moufag Fida, Khalid Alshehri, Baraa Qarah, Baraa Elrahim, Khaled Asiri, Waad Alhawti

**Affiliations:** 1 Preventive Medicine, Saudi Board of Preventive Medicine, Madina, SAU; 2 Health Care Strategy Administration, Madinah Health Cluster, Madinah, SAU; 3 Family Medicine, Taibah University, Madina, SAU; 4 Dentistry, Batterjee Medical College, Jeddah, SAU; 5 Family Medicine, King Abdulaziz University, Jeddah, SAU; 6 Family Medicine, Prince Mishari Bin Saud Hospital, Biljurashi, SAU; 7 Infection Control, Eastern Health Cluster, Dammam, SAU; 8 Family Medicine, Dubai Medical University, Dubai, ARE; 9 Family Medicine, King Khalid University, Abha, SAU; 10 Family Medicine, King Saud University, Riyadh, SAU

**Keywords:** kingdom of saudi arabia (ksa), madinah, prevalence, migraine, depression, anxiety

## Abstract

Introduction: This cross-sectional study addresses the global rise in mental health disorders, emphasizing the bidirectional relationship between migraines and conditions such as depression and anxiety. This study seeks to bridge a crucial gap by examining the prevalence of depression and anxiety and their potential role as predictors of migraine.

Methods: This study included 407 participants aged 16 and above, attending one of the major PHC centers in Madinah city between August 1, 2023, and October 1, 2023. The study employed the Migraine Screening Questionnaire (MS-Q) for migraine screening and the Patient Health Questionnaire-4 (PHQ-4) for identifying anxiety and depression.

Results: Among the 407 participants included in our study, the prevalence of anxiety and depression was 9.1% and 5.9%, respectively. The prevalence of individuals experiencing both anxiety and depression was 3.7%. Anxiety exhibited a robust and statistically significant prediction of having migraines (OR: 4, P<0.05), while depression showed no statistically significant association. Gender, working frequency, and a higher level of education emerged as significant predictors of anxiety. Conversely, working multiple shifts and increased coffee consumption were found to be protective against anxiety. Regarding depression, spending more screen time and a higher education level were identified as significant predictors, while higher coffee intake and current smoking status were protective against depression.

Conclusions: This cross-sectional study concluded that anxiety significantly predicts having migraines, while depression did not emerge as a statistically significant predictor. The study's outcomes underscore the imperative for mental health screening and management in individuals with migraines in PHC settings. However, comprehensive efforts are warranted to be applied across diverse cities and demographics to attain a more nuanced understanding of this association.

## Introduction

Mental health disorders, particularly depression and anxiety, are increasingly being recognized as significant public health concerns worldwide [1]. These conditions not only cause individual suffering but also have an impact on societal well-being and healthcare systems [2]. At the same time, migraine, a common neurological disorder, has gained attention for its possible association with mental health problems [3].

According to the World Health Organization (WHO), depression impacts more than 5% of the global population, with a 50% higher incidence among women than men [4]. Similarly, anxiety currently affects a comparable percentage of the worldwide population, estimated at 4% globally [5]. A comprehensive national study conducted on the general population of Saudi Arabia revealed a prevalence of 12.7% for depression and 12.4% for anxiety [6].

Recent evidence indicates a bidirectional relationship between migraine and mental health disorders [7]. Individuals experiencing depression and anxiety are noted to have an elevated likelihood of encountering migraine, underscoring the importance of gaining a comprehensive understanding of these concurrent conditions.

The primary challenges in psychiatric comorbidity associated with migraines arise from difficulties in controlling excessive worry and achieving a state of relaxation [8]. Additionally, anxiety and depression demonstrate significant independent associations with various aspects of migraines, including frequency, severity, disability, headache impact, quality of life, and sleep quality among individuals experiencing migraines [9].

In a particular study involving 1713 adults identified with migraines, 11.2% experienced depression, 14.6% encountered anxiety, and 13.7% simultaneously grappled with both conditions [10]. A parallel investigation conducted on the population of Makkah city revealed that among individuals with migraines, the prevalence of depression, anxiety, and stress collectively stood at 78.4%, signifying a notably higher rate compared to non-migraine sufferers [11]. Another study focusing on the Saudi population diagnosed with migraines indicated that 73.3% exhibited anxiety and 70.9% presented with symptoms of depression [12].

Global studies make substantial contributions to our comprehension of mental health; however, localized research is imperative for tailoring interventions to specific communities. In Madina City, a conspicuous void exists in mental health research, particularly regarding depression, anxiety, and their correlation with migraines.

This cross-sectional study endeavors to bridge this gap by examining the prevalence of depression and anxiety and exploring their potential association with migraines among prominent PHC centers in Madina City.

## Materials and methods

This is an analytical, multicenter, cross-sectional study. Inclusion criteria encompassed patients of both genders aged 16 and above, attending any of the major PHC centers in Madinah city for any reason between August 1, 2023, and October 1, 2023, and proficient in Arabic. Patients unable to complete the questionnaire or provide informed consent were excluded.

A total of 407 participants were included in the study. The sample size was determined using the formula: n=Z2α P(1-P)/d2. Where n is the sample size, Z is the statistic corresponding to the level of confidence, P is the expected prevalence, and d is precision. A sample size of 180 participants was initially considered sufficient.

Four major PHC centers in Madinah city were equally selected to collect our sample. A systematic sampling approach was employed, including every third patient attending each PHC, to be included in the study.

The Migraine Screening Questionnaire (MS-Q) was utilized to screen PHC attendees for migraines. The MS-Q is a self-administered tool that comprises five questions related to the frequency and characteristics of headaches, as well as the presence or absence of migraine-related symptoms. Each negative response (no) scores 0 points, while each affirmative response (yes) scores 1 point.

The cutoff value for suspecting migraine is set at 4 points or higher, while a score below 4 indicates no suspicion of migraine [13]. Validation of the Arabic version of the MS-Q reported an area under the curve of 0.97 (95% CI, 0.94-0.99), with a sensitivity of 0.95 and a specificity of 0.99 [14].

To identify anxiety and depression, the Patient Health Questionnaire-4 (PHQ-4) was employed. Given the challenges posed by the nature of mood disorders, which can make completing lengthy questionnaires difficult due to fatigue or loss of concentration, we opted for this concise 4-question tool. This tool allows for an ultra-brief and accurate measurement of core symptoms/signs of depression and anxiety by incorporating the two-item measure (PHQ-2), comprising core criteria for depression, and a two-item measure for anxiety (GAD-2). Each question is scored as follows: 0 = not at all; 1 = several days; 2 = more than half the days; 3 = nearly every day. A total score of ≥3 for the first two questions indicates anxiety, while a total score of ≥3 for the last two questions suggests depression. An Arabic version of the PHQ-4 has been validated [15].

To evaluate sociodemographic information, a questionnaire was employed, aiming to ascertain the following details: age, gender, marital status, occupation, working hours, income, education level, hypertension, diabetes, family history of migraine, coffee consumption, smoking habits, type of smoking, daily medication use, screen time, and BMI.

Statistical analysis

Standard software was utilized: SPSS version 21.0 (IBM, Armonk, New York). Data collected from the questionnaires were coded and digitally entered into a spreadsheet for subsequent analysis. The statistical method employed to compare independent variables with the dependent variables was multiple logistic regression.

Ethical considerations

This study adheres to the ethical guidelines outlined by the Declaration of Helsinki. Participants are required to provide informed consent before participating, and all collected data will be treated with confidentiality and anonymity. Ethical approval was obtained from the Institutional Review Board (IRB), General Directorate of Health Affairs in Madinah, NCBE-KACST, KSA: (H-03-M-94).

## Results

A total of 407 individuals from PHC centers were involved in this study, with a nearly equal distribution of genders: 209 males (51%) and 198 females (49%). Approximately 165 participants (41%) were under 26 years old. The marital status of the participants was evenly split between single individuals (184, 45.2%) and married individuals (183, 45.0%). A significant portion of the participants (196, 48.2%) were unemployed at the time of the study. The majority (243, 59.9%) reported working one to six hours per day. About 230 participants (56.6%) reported a monthly income of less than 5000 SAR. In terms of education, 155 participants (38.1%) had achieved a university education or higher (see Table 1). 

**Table 1:** Demographic characteristics of the participants

Characteristics	Frequency (N)	Percent (%)
Gender		
Male	209	51.4
Female	198	48.6
Age		
<18	94	23.1
18-25	71	17.4
26-35	91	22.4
36-45	53	13.0
46-55	36	8.8
>55	62	15.2
Marital status		
Single	184	45.2
Married	183	45.0
Divorced	25	6.1
Widowed	15	3.7
Occupation		
Work in government	92	22.6
Work in private sector	55	13.5
Student	144	35.4
Entrepreneur	23	5.7
Currently, I do not work	64	15.7
Retired	29	7.1
Work shift		
I am not working	196	48.2
Morning shift	68	16.7
Afternoon shift	8	2.0
Evening shift	19	4.7
More than one shift	116	28.5
Daily working hours		
1-6 hours	243	59.9
7-12 hours	160	39.4
>12 hours	3	0.7
Monthly income		
<5000 SAR	230	56.6
5000-10,000 SAR	77	18.9
10000-15000 SAR	68	16.7
>15000 SAR	32	7.9
Educational level		
Primary education	70	17.2
Intermediate education	55	13.5
Secondary education	127	31.2
University level or higher	155	38.1

Table 2 presents the lifestyle and clinical characteristics of the study participants. A significant proportion of participants reported being diagnosed with chronic diseases (141, 35.3%), which is consistent with the expectation of a high prevalence of chronic health conditions among attendees of PHC centers. Regarding coffee consumption, 115 participants (28.3%) reported no intake, while 72 (17.7%) reported consuming more than three cups daily. The prevalence of current smokers was 149 (36.6%), with 240 (59%) reporting as non-smokers and 18 (4.4%) as ex-smokers. 

**Table 2:** Lifestyle and clinical characteristics of the participants

Characteristics	Frequency (N)	Percent (%)
Have you been diagnosed with chronic diseases?		
Yes	141	35.3
No	258	64.7
Coffee consumption per day		
No	115	28.3
1 cup	108	26.5
2 cups	71	17.4
3 cups	41	10.1
>3 cups	72	17.7
Smoking		
Current smoker	149	36.6
Non-smoker	240	59.0
Ex-smoker	18	4.4
Screen time per day		
<2 hours	45	11.1
2-4 hours	135	33.2
4-6 hours	147	36.1
>6 hours	80	19.7

The MS-Q, as outlined in Table 3, delineates the items utilized for screening migraine. In the context of migraine screening using the MS-Q, 121 participants (29.7%) reported experiencing frequent or intense headaches, while 286 (70.3%) indicated "no" and were subsequently excluded from further screening questions. Among those reporting frequent or intense headaches, approximately 57 (47%) reported episodes lasting more than four hours. Furthermore, 59 (48.8%) experienced nausea during headaches, and 99 (81.8%) were sensitive to light or noise during such episodes. The impact of migraines on daily life was assessed, revealing that 77 (63.6%) experienced limitations in physical or intellectual activities due to headaches.

**Table 3:** MS-Q among the participants MS-Q, Migraine Screening Questionnaire

Characteristics	Frequency (N)	Percent (%)

Do you have frequent or intense headaches?		
Yes	121	29.7
No	286	70.3
Do your headaches usually last more than 4 hours?		
Yes	57	47.1
No	64	52.9
Do you usually suffer from nausea when you have a headache?		
Yes	59	48.8
No	62	51.2
Does light or noise bother you when you have a headache?		
Yes	99	81.8
No	22	18.2
Does headache limit any of your physical or intellectual activities?		
Yes	77	63.6
No	44	36.4
MS-Q		
No migraine	54	44.6
Migraine suspected	67	55.4

The results of the MS-Q indicated that among participants experiencing frequent or intense headaches, 67 (55.4%) were classified as having suspected migraines, highlighting the prevalence of this condition among attendees of PHC centers. The calculated migraine prevalence in this study was 16.5%. 

Table 4 displays the distribution of items related to depression and anxiety among the study participants. A significant majority, 323 individuals (79.4%), reported feeling not at all nervous, anxious, or on edge. However, a considerable proportion experienced these feelings on several days (62, 15.2%), more than half the days, or nearly every day (22, 5.4%). Regarding the inability to stop or control worrying, 274 participants (67.3%) reported "not at all," while percentages reporting this concern on several days (87, 21.4%), more than half the days, or nearly every day (46, 11.3%) were notable. Most participants reported not feeling down, depressed, or hopeless at all (337, 82.8%), although a noteworthy percentage experienced these feelings on several days (49, 12.0%). Little interest or pleasure in activities was mostly not reported (334, 82.1%), but a proportion experienced these feelings on several days (51, 12.5%), more than half the days, or nearly every day (22, 5.4%).

**Table 4:** Distribution of the items related to depression and anxiety among the participants using PHQ-4

Items	Frequency (N)	Percent (%)
Feeling nervous, anxious, or on edge		
Not at all	323	79.4
Several days	62	15.2
More than half the days	10	2.5
Nearly every day	12	2.9
Not being able to stop or control worrying		
Not at all	274	67.3
Several days	87	21.4
More than half the days	32	7.9
Nearly every day	14	3.4
Feeling down, depressed, or hopeless		
Not at all	337	82.8
Several days	49	12.0
More than half the days	13	3.2
Nearly every day	8	2.0
Little interest or pleasure in doing things		
Not at all	334	82.1
Several days	51	12.5
More than half the days	12	2.9
Nearly every day	10	2.5
Anxiety prevalence (based on PHQ-4)		
Yes	37	9.1
No	370	90.9
Depression prevalence (based on PHQ-4)		
Yes	24	5.9
No	383	94.1

In terms of the prevalence of anxiety and depression, 9.1% were considered to have anxiety, while 5.9% experienced depression. The prevalence of participants who had both anxiety and depression was 3.7%. 

Table 5 displays the results of the logistic regression model investigating potential predictors of anxiety among the study participants. Age, although not a statistically significant predictor (p=0.105), showed no substantial association with anxiety prevalence. However, a significant gender difference was observed, with females exhibiting significantly higher odds of anxiety compared to males (p=0.002), with an odds ratio of 7.2. Work shift patterns also emerged as influential in anxiety prevalence, particularly for those working more than one shift, showing significantly lower odds of anxiety compared to the unemployed (p=0.001, odds ratio of 0.03). Income level, smoking status, and a history of chronic diseases, on the other hand, were not significant predictors of anxiety.

**Table 5:** Findings of logistic regression model for potential predictors of anxiety among the participants *P-value <0.05 is statistically significant.

Predictors	Categories	Reference group	P-value	Odds ratio	Lower limit (95% CI)	Upper limit (95% CI)
Age	A higher age group	A lower age group	0.105	0.68	0.43	1.08
Gender	Female	Male	0.002*	7.20	2.02	25.67
Work shift	Morning shift	Unemployed	0.074	0.20	0.03	1.17
	Afternoon shift	Unemployed	0.999	0.00	0.01	-
	Evening shift	Unemployed	0.226	0.19	0.01	2.84
	More than one shift	Unemployed	0.001*	0.03	0.01	0.24
Income	A high level of income	A low level of income	0.104	1.62	0.91	2.91
Educational level	A high level of education	A low level of education	0.005*	3.13	1.42	6.89
Coffee consumption per day	More cups	Less cups	0.026*	0.64	0.44	0.95
Smoking	Current smoker	Non-smoker	0.167	0.44	0.14	1.41
	Current smoker	Ex-smoker	0.153	0.15	0.01	2.04
Daily working hours	More time	Less time	0.004*	1.32	1.09	1.58
Chronic diseases	Yes	No	0.108	2.53	0.82	7.87

Educational level showed significance, indicating that a higher level of education was associated with increased odds of anxiety (p=0.005, odds ratio of 3.13). Daily working hours also played a role, with longer hours significantly predicting anxiety (p=0.004, odds ratio of 1.32). Conversely, a higher consumption of cups of coffee was associated with lower odds of anxiety (p=0.026, odds ratio of 0.64).

Table 6 provides insights into potential predictors of depression among the study participants. Although higher age groups demonstrated a trend toward reduced odds of depression, statistical significance was not reached (p=0.061). Similarly, gender did not achieve statistical significance (p=0.090), although it suggested higher odds of depression among females, indicating a potential gender-related trend. Educational background emerged as a significant predictor, with a higher level of education associated with approximately 3.8 times higher odds of having depression (p=0.011). Furthermore, spending more time on screens was associated with 2.3 times higher odds of having depression compared to less screen time (p<0.05).

**Table 6:** Findings of logistic regression model for potential predictors of depression among the participants *P-value <0.05 is statistically significant.

Predictors	Categories	Reference group	P-value	Odds ratio	Lower limit (95% CI)	Upper limit (95% CI)
Age	A higher age group	A lower age group	0.061	0.56	0.30	1.03
Gender	Female	Male	0.090	3.37	0.83	13.71
Educational level	A high level of education	A low level of education	0.011*	3.76	1.35	10.52
Coffee consumption per day	More cups	Fewer cups	0.017*	0.55	0.33	0.90
Smoking	Current smoker	Non-smoker	0.030*	0.22	0.06	0.86
	Current smoker	Ex-smoker	0.584	0.46	0.03	7.55
BMI	A higher value	A lower value	0.258	0.93	0.83	1.05
Screen time	More time	Less time	0.018*	2.33	1.16	4.69
Daily working hours	More time	Less time	0.063	1.15	0.99	1.34

Moreover, higher coffee consumption (p=0.017, odds ratio of 0.55) and being a current smoker (p=0.030, odds ratio of 0.22) were associated with lower odds of depression. These findings enhance our understanding of the factors contributing to the prevalence of depression among PHC attendees in Madinah, highlighting the interplay of age, gender, education, and lifestyle choices in shaping the mental health landscape of this population. All other predictors mentioned in Table 1 and Table 2 were included in the regression analysis but did not show significant associations and were, therefore, excluded through step-back regression analysis.

Table 7 displays the results of the logistic regression model investigating the potential association of anxiety and depression with migraine among the study participants. Participants with anxiety showed a strong and significant association with migraine, displaying approximately a four times increase in the odds of having migraine (p=0.001). Conversely, while depression demonstrated an association with migraine, it did not reach statistical significance (p=0.111). 

**Table 7:** Findings of logistic regression model for potential association of anxiety and depression with migraine *P-value <0.05 is statistically significant.

Predictors	Categories	Reference group	P-value	Odds ratio	Lower limit (95% CI)	Upper limit (95% CI)
Anxiety disorder	Anxiety	No anxiety	0.001*	3.99	1.76	9.02
Depression disorder	Depression	No depression	0.111	2.27	0.83	6.21

## Discussion

The primary aim of this cross-sectional study was to estimate the prevalence of anxiety and depression and explore their association with migraine in the population of Madina city, Saudi Arabia. Our findings revealed that 9.1% of individuals attending PHC centers in Madina city experienced anxiety, 5.9% reported symptoms of depression, and 3.7% had both anxiety and depression. Compared to other regions in Saudi Arabia, the prevalence of anxiety and depression in Madina city appears lower than the local prevalence of anxiety (12.4%) and depression (12.7%) [6]. This discrepancy could potentially be influenced by the spiritual comfort provided by the presence of the holy mosque of the Prophet Mohammed among Muslims. However, further investigation is required to confirm this relationship, which is beyond the scope of our study.

One study characterized the severity of depression and anxiety among the general population of Madina city as mild and moderate, respectively [14]. In contrast, our study found that a significant majority (79.4%) reported feeling not at all nervous, but a considerable proportion experienced these feelings on more than half the days or nearly every day (5.4%), indicating a more severe level of anxiety. Similarly, while the majority reported not feeling down, depressed, or hopeless at all (82.8%), a noteworthy percentage experienced these feelings more than half the day or nearly every day (5.4%), indicating a more severe level of depression among this group of participants.

A significant predictor of anxiety in our study was female gender, with females being 7.2 times more likely to experience anxiety compared to males (p<0.05). This gender-based variation is consistent with another study that concluded that anxiety disorders are not only more common but also more incapacitating in females compared to males [15]. Income level, smoking, and a history of chronic diseases, on the other hand, were not significant predictors of anxiety, contradicting other studies that found significant associations among them and with anxiety [16]. This discrepancy could be due to differences in demographic characteristics and sample sizes.

Coffee consumption was identified as a protective factor against anxiety in our study, contradicting other studies that showed a positive correlation between coffee consumption and anxiety levels [17]. This discrepancy could be attributed to the type of coffee typically consumed by our population, which includes Arabic coffee in small quantities.

Regarding depression, significant predictors included a higher level of education, with approximately 3.8 times higher odds of having depression compared to a lower level of education (p<0.05) and spending more time on screens, which was associated with 2.3 times higher odds of having depression compared to less screen time (p<0.05). This result aligns with research suggesting that spending more than four hours per day watching TV and using computers correlates with moderate to severe levels of depression [18]. Further studies are needed to classify the types of screens, including smartphones, TVs, or video games.

In our study, anxiety was significantly associated with migraine, whereas depression did not reach statistical significance in its association with migraine. Previous studies have observed a reciprocal relationship between depression and migraine, with depression being identified as a significant predictor of migraine [19]. This finding contradicts the results of our study, which did not find depression to be a significant predictor of migraine. However, anxiety has been reported in multiple national similar studies as one of the main triggers for migraine attacks [20,21]. This aligns with our finding that participants with anxiety exhibited a strong and significant association with migraine, showing approximately four times higher odds of having migraine (p=0.001).

From a public health perspective, identifying these mental disorders among the studied population highlights their significant societal impact, underscoring the need for resource allocation and strategic planning. In clinical practice, healthcare professionals should remain vigilant regarding the prevalence of anxiety and depression, adopting a comprehensive assessment and management approach.

Researchers can use these findings as a foundation to explore the underlying mechanisms of anxiety and depression, along with their connection to migraine. This includes investigating molecular and genetic factors contributing to these conditions and understanding their longitudinal progression. Future studies might focus on developing targeted treatments to enhance the quality of life for affected individuals and improve overall outcomes.

While this research on anxiety and depression prevalence and their association with migraine is enlightening, it is essential to acknowledge potential limitations. The reliance on self-reported data and a cross-sectional design may introduce recall bias and hinder the establishment of causal relationships. Additionally, potential sampling bias and limited generalizability might impact the external validity and applicability of the study's findings.

## Conclusions

This study sheds light on the interplay between anxiety, depression, and migraine among individuals attending PHC centers in Madina City. The findings underscore the importance of screening and managing mental health conditions in this population to enhance overall well-being. While the study emphasizes the need for targeted interventions, future research with larger and diverse samples in various cities is recommended to deepen our understanding of these relationships and develop more effective intervention protocols. The results contribute to the broader effort of improving mental health care in different demographics, ultimately benefiting individuals experiencing these interconnected health concerns.
